# PML Alternative Splice Products Differentially Regulate HAdV Productive Infection

**DOI:** 10.1128/spectrum.00785-22

**Published:** 2022-06-14

**Authors:** Julia Mai, Miona Stubbe, Samuel Hofmann, Sawinee Masser, Thomas Dobner, Christopher Boutell, Peter Groitl, Sabrina Schreiner

**Affiliations:** a Institute of Virology, Hannover Medical School, Hannover, Germany; b Institute of Virology, School of Medicine, Technical University of Munich, Munich, Germany; c MRC-University of Glasgow Centre for Virus Research (CVR), Glasgow, Scotland, United Kingdom; d Heinrich-Pette-Institute, Leibniz Institute for Experimental Virology, Hamburg, Germany; e Institute of Virology, Helmholtz Zentrum München, Munich, Germany; f Cluster of Excellence RESIST (Resolving Infection Susceptibility; EXC 2155), Hannover Medical School, Hannover, Germany; University of Arizona

**Keywords:** human adenovirus, HAdV, PML-NB, SUMO

## Abstract

Promyelocytic leukemia nuclear bodies (PML-NBs) were considered to maintain antiviral capacity, as these spherical complexes are antagonized by viruses. Actual work provides evidence, that PML-NB-associated factors might also be beneficial for distinct viral processes indicating why genomes and replication centers of nuclear replicating viruses are often found juxtaposed to PML-NBs. Several early HAdV proteins target PML-NBs, such as E4orf3 that promotes redistribution into track-like structures. PML-associated dependency factors that enhance viral gene expression, such as Sp100A remain in the nuclear tracks while restrictive factors, such as Daxx, are inhibited by either proteasomal degradation or relocalization to repress antiviral functions. Here, we did a comprehensive analysis of nuclear PML isoforms during HAdV infection. Our results show cell line specific differences as PML isoforms differentially regulate productive HAdV replication and progeny production. Here, we identified PML-II as a dependency factor that supports viral progeny production, while PML-III and PML-IV suppress viral replication. In contrast, we identified PML-I as a positive regulator and PML-V as a restrictive factor during HAdV infection. Solely PML-VI was shown to repress adenoviral progeny production in both model systems. We showed for the first time, that HAdV can reorganize PML-NBs that contain PML isoforms other then PML-II. Intriguingly, HAdV was not able to fully disrupt PML-NBs composed out of the PML isoforms that inhibit viral replication, while PML-NBs composed out of PML isoforms with beneficial influence on the virus formed tracks in all examined cells. In sum, our findings clearly illustrate the crucial role of PML-track formation in efficient viral replication.

**IMPORTANCE** Actual work provides evidence that PML-NB-associated factors might also be beneficial for distinct viral processes indicating why genomes and replication centers of nuclear replicating viruses are often found juxtaposed to PML-NBs. Alternatively spliced PML isoforms I-VII are expressed from one single *pml* gene containing nine exons and their transcription is tightly controlled and stimulated by interferons and p53. Several early HAdV proteins target PML-NBs, such as E4orf3, promoting redistribution into track-like structures. Our comprehensive studies indicate a diverging role of PML isoforms throughout the course of productive HAdV infection in either stably transformed human lung (H1299) or liver (HepG2) cells, in which we observed a multivalent regulation of HAdV by all six PML isoforms. PML-I and PML-II support HAdV-mediated track formation and efficient formation of viral replication centers, thus promoting HAdV productive infection. Simultaneously, PML-III, -IV,-V, and -VI antagonize viral gene expression and particle production.

## INTRODUCTION

Promyelocytic leukemia nuclear bodies (PML-NBs) are multiprotein complexes with an average size of 0.2 to 1.0 μm. They are matrix-associated dynamic structures that harbor more than 166 proteins ether constitutively or transiently, depending on different conditions such as DNA repair, oncogenic transformation, stress response, interferon expression, senescence, apoptosis or also viral infections (1–7). Main constitutive components of PML-NBs except of the tumor suppressor PML are the transcriptional modulator Sp100 (Speckled protein 100 kDa), the chromatin remodeling factor Daxx (Death domain-associated protein 6), the Bloom Helicase (BLM), and the small ubiquitin-like modifier (SUMO) (8–10). These nuclear dot-like structures were first identified in acute promyelocytic leukemia (APL) patients, where the PML gene is fused to the retinoic acid receptor α (RARα) gene due to the consistent chromosomal translocation t(15:17) (11), resulting in the disruption of the PML-NBs by the oncogenic PML-RARα fusion protein (3).

Seven PML isoforms (PML-I to PML-VII) with a weight range of 48 to 97 kDa are expressed by alternative splicing from one single *pml* gene containing nine exons (12). The transcription is tightly controlled and stimulated by interferon due to a 5′ IFN-alpha/-beta stimulated response element (ISRE) and an IFN-gamma activation site (GAS) in the untranslated first exon of the *pml* gene (13). Additionally, the tumor suppressor p53 binds to the *pml* gene inducing the mRNA expression of PML, while the *pml* gene can act upstream of p53 to enhance p53 transcription and downstream to promote antiproliferative functions of p53 (14). All PML isoforms share the same N-terminal part consisting of a the RBCC/TRIM motif, which affects apoptotic and antiviral activities due to PML protein-protein interactions (12). Despite the RBCC motif, all PML isoforms contain two SUMO conjugating motifs (SCM) in the N-terminal part. PML-I to PML-VI contain an additional SCM and a nuclear localization signal (NLS). Furthermore, a SUMO interacting motif (SIM) is located in PML-I to PML-V (15–17).

Posttranslational modification of PML and PML-NB associated proteins by the small ubiquitin-like protein SUMO plays an integral role in regulating PML-NB functions, integrity, and formation (2, 18). Both the covalent conjugation of SUMO and the non-covalent interaction of SUMO with the SUMO interacting motif (SIM) of PML and associated proteins are required for formation and function of PML-NB (19, 20). Formation starts with dimerization of the PML proteins via their RBCC motif at the N-terminal part prior to multimerization by nucleation and maturation of nuclear bodies through SUMOylation of PML proteins and recruitment of SIM-containing or SUMOylated partners (or both) by the SUMO or SIM of PML into the inner core of the spherical bodies (3, 19). Since the discovery of PML-NBs, these multiprotein complexes were considered to be involved in antiviral mechanisms (21, 22). This was proposed, as their main body components are *interferon-stimulated genes* (ISGs) that efficiently impede viral infections (23–25). Simultaneously, PML-NBs are targeted by a wide array of DNA and RNA viruses to counteract viral defense mediated by NB associated factors (26).

Recently, we gained knowledge showing that several PML-NB factors are essential to support virus replication and productive infection, explaining why the viral genomes of HAdV are found juxtaposed to PML-NBs (27, 28). Several early HAdV proteins target PML-NBs (28). In detail, E4orf3 is known to specifically interact with PML-II (29). In addition, E4orf3 promotes reorganization of the nuclear bodies into track-like structures by multimerization of the viral protein (30, 31). SUMO modification of the HAdV DNA binding protein E2A mediates the juxtaposed localization of PML tracks and viral replication compartments (27). Dependency factors that are identified to promote viral replication, such as Sp100A, remain associated with the nuclear tracks supporting viral transcription, whereas negative factors, such as Daxx, ATRX, p53, and Sp100B are either targeted for ubiquitin-dependent proteasomal degradation via (i) E1B-55K alone, (ii) E1B-55K/E4orf6 mediated E3 ubiquitin ligases or (iii) proteolysis involving the host SUMO-dependent ubiquitin ligase RNF4, or (iv) relocalization to the inner fraction of replication centers (32–39). Furthermore, E1B-55K interacts with PML-IV and PML-V in a SUMO-dependent and -independent manner. Interaction with PML-IV mediates the localization of E1B-55K and p53 to PML-NBs (40, 41). Additionally, E1A-mediated transactivation of viral transcription is promoted by interaction of PML-II with E1A-13S (32).

Reflecting on our data generated during HAdV infection analyzing the role of Sp100 alternatively spliced isoforms A, B, C, and HMG, we realized that splice variants might constitute opposing functions during HAdV infection and either support or block viral gene expression events (33). So far, it has only been reported that PML-II promotes E1A mediated HAdV transcription (32), whereas the fate and function of the other PML splice variants during infection is not investigated. Moreover, our group recently showed that the therapeutic agent, arsenic trioxide (ATO), efficiently inhibits HAdV infection by modulation of PML-NBs, emphasizing the importance of complete understanding of the PML role in infection (42). Therefore, we aimed to investigate the influence of each specific PML isoform alone on the HAdV infection processes.

Our comprehensive studies indicate a diverging role of PML isoforms throughout the course of productive HAdV infection in either stably transformed human lung (H1299) or liver (HepG2) cells, in which we observed a multivalent regulation of HAdV by all six PML isoforms. PML-I and PML-II support HAdV-mediated track formation and efficient formation of viral replication center formation, thus promoting HAdV productive infection. Simultaneously, PML-III, -IV, -V, and -VI antagonize viral gene expression and particle production.

## RESULTS

### Protein levels of PML isoforms are differentially regulated in HAdV infected cells.

**FIG 1:**
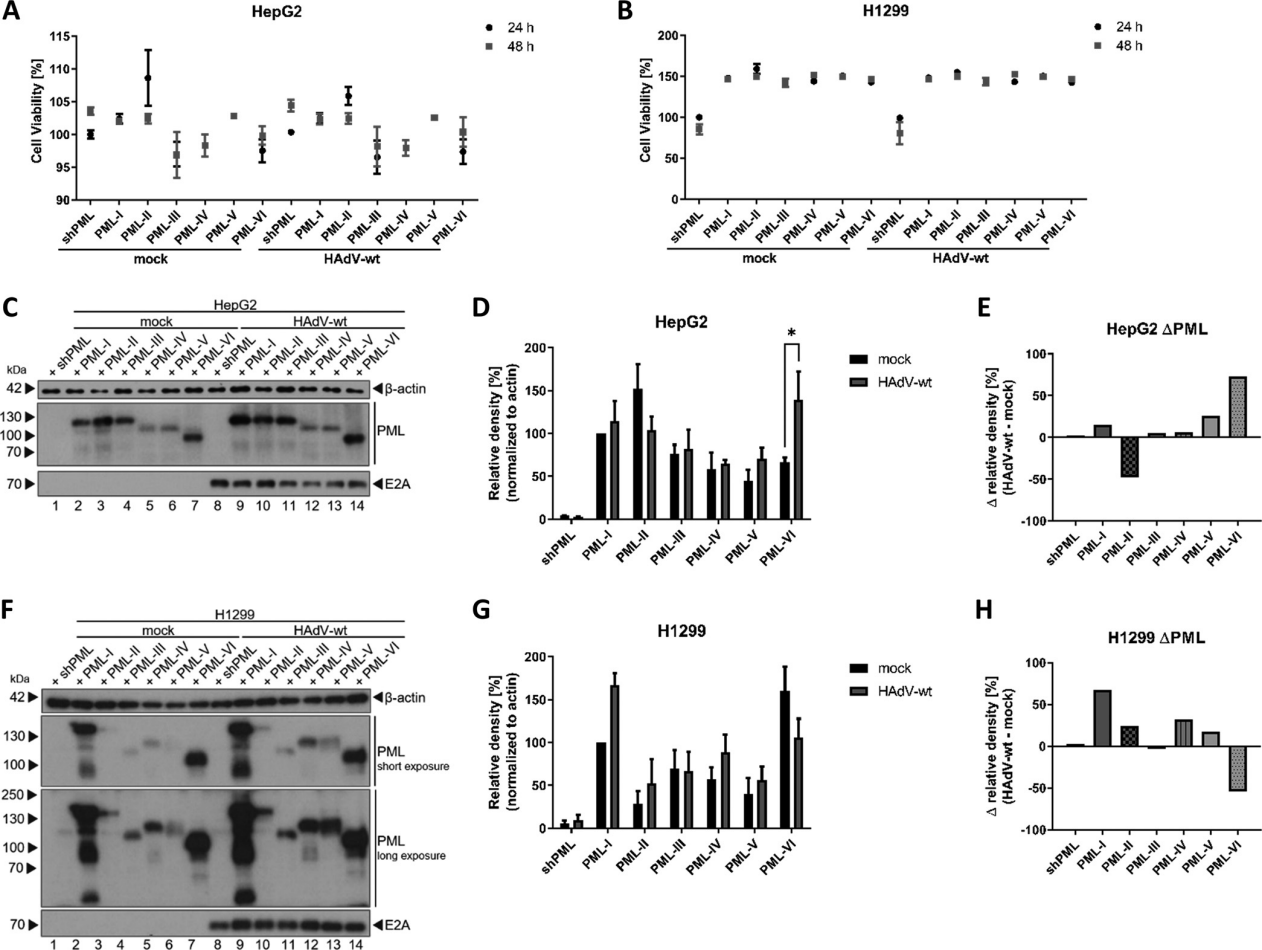
HAdV-wt promotes PML isoform expression. (A) HepG2 shPML and EYFP-PML.n cells and (B) H1299 shPML and EYFP-PML.n cells were infected with HAdV-wt at a multiplicity of 50 FFU/cell or 20 FFU/cell, respectively. Cell viability was determined using the CellTiter-Blue Cell viability system and measured using a Tecan Infinite 200M plate reader 25 h and 48 h p.i. *xy* charts represent average values and standard deviations based on three independent experiments. (C) HepG2 shPML and EYFP-PML.n cells and (F) H1299 shPML and EYFP-PML.n cells were infected with HAdV-wt at a multiplicity of 50 FFU/cell or 20 FFU/cell, respectively. (C, F) Cells were harvested 24 h p.i. and total-cell lysates were prepared. Total-cell lysates were separated by SDS-PAGE and subjected to immunoblot analyses using pAb rabbit α-PML (anti-PML), MAb mouse B6-8 (anti-E2A), and MAb mouse AC-15 (anti-β-actin). Molecular weights in kDa are indicated on the left, relevant proteins on the right. Densitometric analyses of PML isoform levels in HepG2 shPML/EYFP-PML.n (D) and in H1299 shPML/EYFP-PML.n (G), quantified with *ImageJ* (version 1.51f) software and normalized to respective anti-β-actin levels. Bar charts represent average values and standard deviations based on three independent experiments for HepG2 cells and four independent experiments for H1299 cells. Statistically significant differences were assessed using two-way ANOVA and Šidák correction test with the GraphPad Prism5 software. *, *P* ≤ 0.05; **, *P* ≤ 0.01; ***, *P* ≤ 0.001; ****, *P* ≤ 0.0001. Calculated expressions levels of PML isoforms in HAdV-wt infected cells were subtracted from the corresponding mock samples to illustrate PML protein level regulation by HAdV-wt. Bar charts represent delta values of the single PML isoforms in HepG2 (E) and H1299 (H) cells.

Based on our previous observations, PML is involved in antiviral responses as a stable PML knock-down shows a moderate increase in viral progeny production (32). To further reveal the role of each distinct PML isoform in HAdV regulation, we stably transduced human hepatocellular carcinoma (HepG2) and human lung carcinoma (H1299) cells with a lentiviral vector encoding PML specific shRNA sequence for efficient depletion of all endogenous PML isoforms. To then reintroduce each single PML isoform, EYFP-tagged PML isoforms resistant to the shRNA were transduced into the cells lacking PML expression (43). To first monitor growth behavior of these newly generated cell lines and potential influence of each PML isoform, we monitored cell viability and no strong effect was observed among the not infected cell lines and the HAdV infected samples (Fig. 1A, B).

Then, cells were infected with HAdV-wt, and PML isoform protein levels were determined by Western blotting and compared to uninfected approaches (Fig. 1C, F). No significant change in PML protein levels was observed in HepG2 cells expressing PML-I, -II, -III, -IV, and -V. However, PML-VI protein levels were significantly increased by 73.1% after wt infection compared with uninfected HepG2 control cells (Fig. 1D, E). In H1299 cells, protein levels of PML isoforms were not significantly changed during HAdV-wt infection (Fig. 1G, H).

The molecular weight of PML-III differs between the two cell lines. In HepG2 cells PML-III was detected at a height of around 120 kDa (Fig. 1C), while in H1299 cells, PML-III was detected at a height of 100 kDa adequate to an EYFP-tagged version of PML-III (Fig. 1F).

### PML isoforms modulate HAdV progeny production.

**FIG 2:**
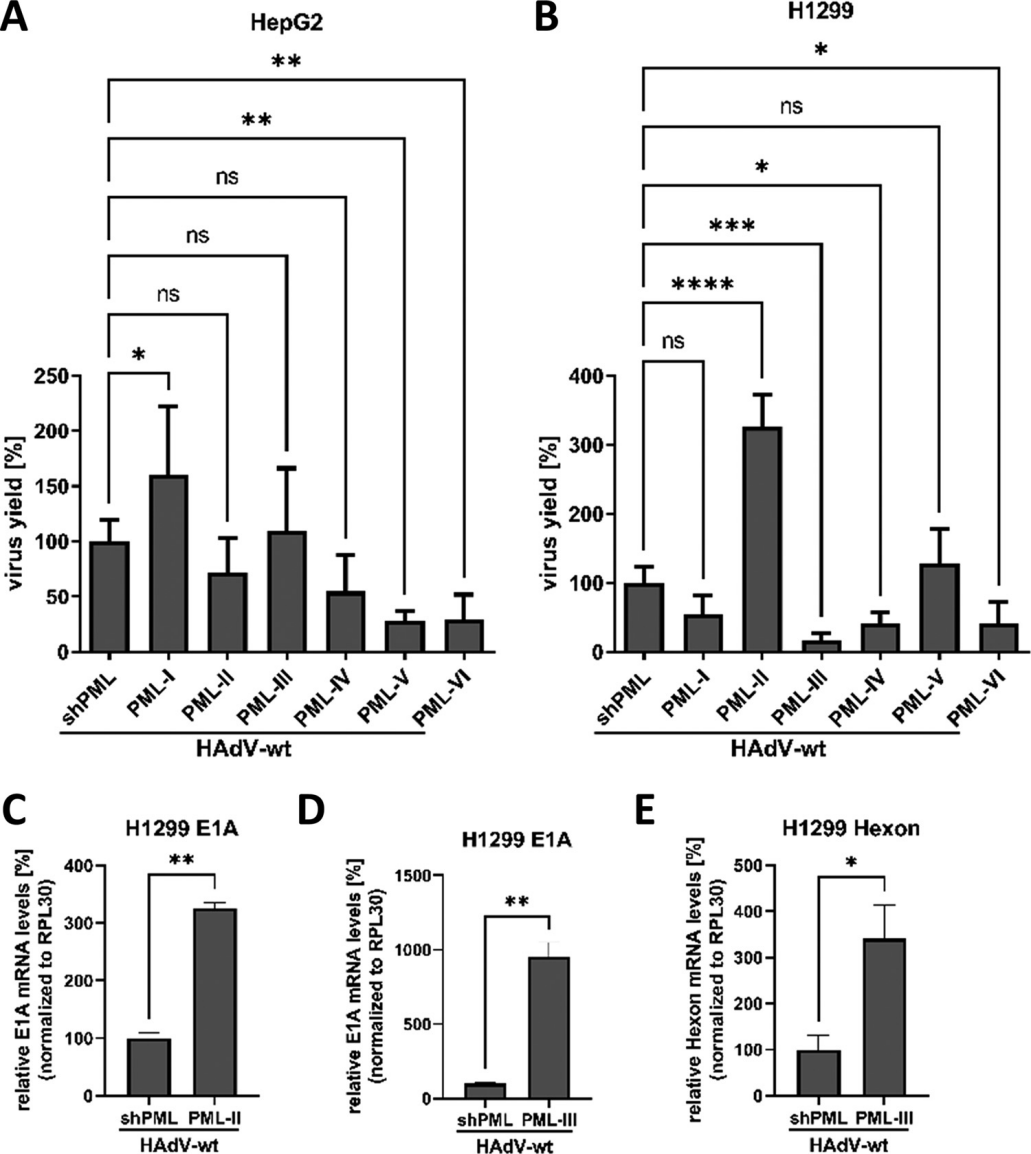
PML differentially affect HAdV-wt replication. (A) HepG2 shPML/EYFP-PML.n cells and (B, C, D, E) H1299 shPML/EYFP-PML.n cells were infected with HAdV-wt at a multiplicity of 50 FFU/cell or 20 FFU/cell, respectively, and harvested 24 h p.i. (A, B) Virus particles were isolated from infected cells. and viral progeny production was determined using quantitative immunofluorescence staining of E2A/DBP in HEK293 cells. Bar charts represent average values and standard deviations based on two independent experiments. Statistically significant differences were assessed using one-way ANOVA and Dunnett’s test with the GraphPad Prism5 software. *, *P* ≤ 0.05; **, *P* ≤ 0.01; ***, *P*≤ 0.001; ****, *P* ≤ 0.0001. (C, D, E) Total mRNA was isolated using TRIzol, reverse transcribed and analyzed by RT-qPCR using primers specific for HAdV E1A (C, D) and hexon (E). The data was normalized to the respective RPL30 mRNA levels. Bar charts represent average values and standard deviations based on two independent experiments measured in technical duplicates. Statistically significant differences were assessed using an unpaired Student’s *t* test with the GraphPad Prism9 software. *, *P* ≤ 0.05; **, *P* ≤ 0.01; ***, *P* ≤ 0.001; ****, *P* ≤ 0.0001.

To elucidate the influence of PML isoforms on HAdV life cycle, we investigated viral progeny production, DNA synthesis, and gene expression in the presence of each PML isoform. We infected transduced HepG2 shPML/EYFP-PML.n and H1299 shPML/EYFP-PML.n cells and isolated newly synthesized virions, DNA, and mRNA from each sample to analyze the impact of PML isoforms on viral replication. Expression of specific PML isoforms showed diverse effects on viral progeny production in infected HepG2 shPML/EYFP-PML.n cells. PML-I led to a 30.5% increase in viral progeny production compared to shPML control cells. Compared with infected shPML HepG2 cells (Table 1), the expression of PML-V reduced the viral progeny production by 73.3%, while PML-VI expression even led to a 77.1% decrease (Fig. 2A). PML-II, -III, and -IV did not significantly affect viral progeny production in HepG2 cells (Fig. 2A).

**TABLE 1:** PML isoform mediated regulation of HAdV infection in HepG2 cells^*a*^

HepG2	PML-I	PML-II	PML-III	PML-IV	PML-V	PML-VI
Progeny production	↑	ns	ns	ns	↓	↓
HAdV DNA synthesis	ns	ns	ns	ns	ns	ns
HAdV mRNA expression	ns	ns	ns	ns	ns	ns
E1A	ns	ns	ns	ns	ns	ns
E1B-55K	ns	ns	↓	↓	ns	ns
E2A	↓	↓	↓	↓	ns	ns
E4orf6	↓	↓	↓	↓	↓	↓
E4orf6/7	↑	↑	ns	ns	ns	ns
E4orf3	ns	ns	ns	↓	↓	ns
IVa2	ns	↓	↓	↓	↓	↓
pVI	ns	ns	↓	↓	ns	ns
L4-33K	ns	↓	↓	↓	ns	ns
L4-100K	ns	↓	↓	↓	↓	↓
Capsid	ns	ns	↓	↓	ns	ns
Capsid-associated	ns	↑	ns	ns	↑	ns
HAdV RC formation	ns	ns	ns	ns	ns	ns
PML tracks formation	yes	yes	yes/no	yes	yes/no	yes/no

a Effects of PML isoforms on HAdV progeny production, DNA synthesis, mRNA, and protein expression and RC formation are represented with following symbols: ↑, promoting effect; ↓, inhibitory effect; ns, no effect. The formation of PML tracks is labeled with “yes,” the lack of reorganization into PML tracks with “no,” and mixed phenotype with “yes/no.”

Compared with infected shPML H1299 cells (Table 2), viral progeny production was increased by 238% in EYFP-PML.II cells (Fig. 2B). Moreover, our data revealed the negative influence of PML isoforms -III, -IV, and -VI on viral progeny production in H1299 cells. The expression of PML-III significantly reduced viral progeny production by 83.7%, PML-IV induced 58% reduction, and PML-VI even led to 60% reduction in viral progeny production compared with shPML H1299 cells (Fig. 2B). PML isoforms -I and -V did not significantly change the viral progeny production in H1299 cells (Fig. 2B).

**TABLE 2:** PML isoform mediated regulation of HAdV infection in H1299 cells^*a*^

H1299	PML-I	PML-II	PML-III	PML-IV	PML-V	PML-VI
Progeny production	ns	↑	↓	↓	ns	↓
HAdV DNA synthesis	ns	ns	ns	ns	ns	ns
HAdV mRNA expression	ns	↑	↑	ns	ns	ns
E1A	↑	ns	↑	ns	ns	ns
E1B-55K	ns	ns	↑	ns	ns	ns
E2A	↓	↓	↓	↓	ns	ns
E4orf6	↓	ns	ns	ns	ns	ns
E4orf6/7	ns	ns	ns	ns	ns	ns
E4orf3	ns	ns	ns	ns	ns	ns
IVa2	ns	ns	ns	ns	ns	↓
pVI	ns	↑	↑	ns	ns	ns
L4-33K	ns	↓	ns	ns	ns	ns
L4-100K	ns	↑	↑	↑	ns	↑
Capsid	↓	ns	ns	ns	ns	ns
Capsid-associated	↓	↓	ns	ns	↓	↓
HAdV RC formation	ns	ns	ns	ns	↓	↓
PML tracks formation	yes	yes	yes/no	yes	yes/no	yes/no

a Effects of PML isoforms on HAdV progeny production, DNA synthesis, mRNA, and protein expression and RC formation are represented with following symbols: ↑, promoting effect; ↓, inhibitory effect; ns, no effect. The symbol “↑↓” was used to represent both promoting and inhibitory effect on the expression of different proteins. The formation of PML tracks is labeled with “yes,” the lack of reorganization into PML tracks with “no,” and mixed phenotype with “yes/no.”

### PML isoforms affect mRNA synthesis.

HAdVs position their genomes next to PML-NBs and *de-novo* assembly of PML-NBs takes place juxtaposed to the sites of adenoviral replication (22, 26). Here, we isolated viral and genomic DNA from HepG2 shPML/EYFP-PML.n and H1299 shPML/EYFP-PML.n cells and quantified viral genome copies by qPCR using specific primers for the viral coding region of the *hexon* gene. Relative viral DNA copies in HepG2 and H1299 cells expressing single PML isoforms were analyzed in comparison with cells depleted for endogenous PML. We did not observe any significant changes in viral DNA synthesis after expression of single PML isoforms (data not shown).

PML isoform-specific regulation of viral mRNA expression was then examined performing RT-qPCR for viral early E1A and late Hexon mRNA. Because transcription of certain host genes can be regulated by PML isoforms, we tested the different housekeeping genes RPL30 (Fig. S1A), 18S (Fig. S1B), CD45 (Fig. S1C), and IQGAP3 (Fig. S1D). We proceeded our further analyses with primers specific for RPL30 because gene transcription of RPL30 was shown to be highly similar among the different cell lines (Fig. S1A). In HepG2 cells viral mRNA was only moderately regulated by PML isoforms with no significant changes (data not shown) (Table 1). On the other hand, we observed significant increase of viral E1A mRNA by 225.8% and 854.4% after expression of PML-II (Fig. 2C) and PML-III (Fig. 2D), respectively. Moreover, Hexon mRNA production was significantly enhanced in PML-III expressing H1299 cells by 241.4% (Table 2) (Fig. 2E).

### PML isoforms regulate early viral protein expression and splicing patterns.

**FIG 3:**
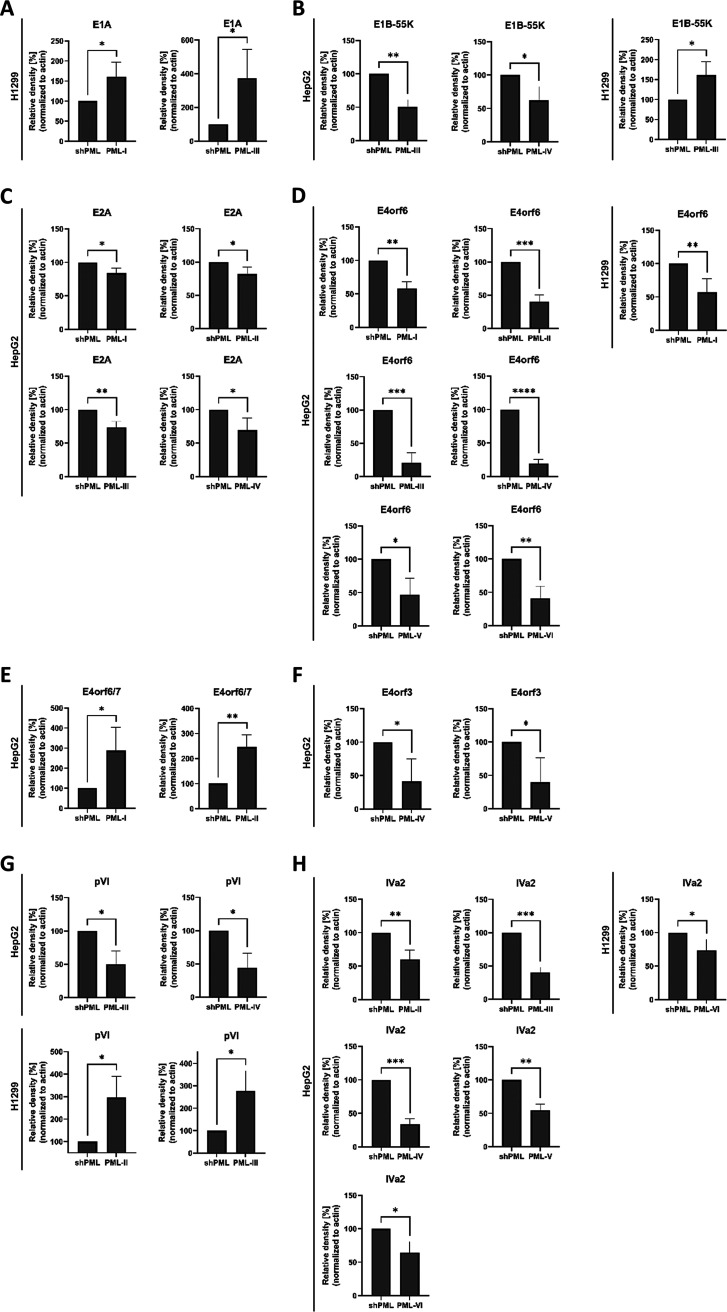
Expression of early viral proteins and the cement protein pVI differs after expression of single PML isoforms. HepG2 shPML/EYFP-PML.n and H1299 shPML/EYFP-PML.n cells were infected with HAdV-wt at a multiplicity of 50 FFU/cell or 20 FFU/cell, respectively, and harvested 24h p.i. Total-cell lysates were prepared and proteins were separated using SDS-PAGE and detected via immunoblotting using M73 (anti-E1A), 2A6 (anti-E1B-55K), B6-8 (anti-E2A), RSA3 (anti-E4orf6), 6A11 (anti-E4orf3), anti-pVI, anti-IVa2, and AC-15 (anti-β-actin). Molecular weights in kDa are indicated on the left, relevant proteins on the right. (B) Densitometric analyses of detected protein levels quantified with *ImageJ* (version 1.51f) software and normalized to respective α-β-actin steady state levels. Bar charts represent average values and standard deviations based on three independent experiments in HepG2 cells and on four independent experiments for H1299 cells for (A) E1A, (B) E1B-55K, (C) E2A, (D) E4orf6, (E) E4orf6/7, (F) E4orf3, (G) pVI, and (H) IVa2. Statistically significant differences were assessed using an unpaired Student's *t* test with the GraphPad Prism9 software. *, *P* ≤ 0.05; **, *P* ≤ 0.01; ***, *P* ≤ 0.001; ****, *P* ≤ 0.0001.

PML proteins are capable to bind to and/or to promote transcriptional repression of cellular and viral factors, while also regulating proteasomal protein degradation processes in the cell (33, 40, 41, 44–46). To elucidate the effect of PML isoforms on HAdV protein levels, we performed Western blot analysis of infected HepG2 shPML/EYFP-PML.n (Table 1) and H1299 shPML/EYFP-PML.n (Table 2) (Fig. 3) cells. E1A is the first protein expressed during HAdV infection and known to deregulate the host cell cycle by pRb inactivation and release of E2F (47, 48). The 13S isoform of the oncoprotein E1A was shown to specifically interact with PML-II promoting the E1A-induced transactivation of viral gene expression (32).

In H1299 cells, E1A protein levels were shown to be significantly increased by isoforms PML-I and PML-III. Here E1A was induced by 45.1% and by 274.4% after PML-I and PML-III expression, respectively (Fig. 3A).

E1B-55K is a multifunctional protein involved in the viral life cycle (49–54). E1B-55K interacts with PML-IV and PML-V in a SUMO-dependent manner mediating SUMOylation of the PML-NB component p53 and thereby contributing to oncogenic transformation (28, 40). In HepG2 cells, exclusive expression of PML-I, PML-II, PML-V, and PML-VI did not affect E1B-55K protein levels, but we observed a significant decrease when PML-III or PML-IV were expressed, with E1B-55K protein levels decreased by 49.6% or by 38%, respectively (Fig. 3B).

For lung carcinoma H1299 cells, we only observed moderate PML isoform-specific changes of E1B-55K levels. Namely, PML-III, increased E1B-55K levels by 61% in comparison with shPML cells (Fig. 3B).

E2A/DBP is considered a marker for replication centers and is further implicated in various processes, such as virion assembly, viral transcription, cellular transformation and mRNA stability (55–63). We reported that E2A SUMOylation is required for the localization of viral genomes next to PML-NBs. Furthermore, reduced SUMOylation of E2A lowers the amount of replication centers and PML-NBs (27). We observed that in HepG2 cells expression of PML-I, PML-II, PML-III, and PML-IV reduces E2A levels by 16.2%, 18.2%, 26.5%, or 30.8%, respectively. On the other hand, E2A expression in H1299 cells was not significantly changed in cells expressing individual PML isoforms in comparison to shPML cells (Fig. 3C).

E4orf3 or E4orf6 are necessary to ensure efficient viral replication and share redundant roles in late protein synthesis, late viral mRNA transport and progeny virus production (29, 56, 64–70). E4orf6 is involved in formation of the Cullin-based E3 ubiquitin ligase complex with E1B-55K that targets host cell factors for proteasomal degradation (34). E4orf6/7 protein complexes with the E2F transcription factor thereby inducing adenoviral transcription from the E2 promoter (71). Here, we report that PML isoforms have a strong impact on E4orf6 protein levels in HepG2 cells with a significant decrease in viral expression compared with cells depleted for endogenous PML (Fig. 3D). E4orf6 levels were reduced 42.1% by PML-I, 59.3% by PML-II, 79.3% by PML-III, 80.5% by PML-IV, 53.3% by PML-V, and 58.7% by PML-VI (Fig. 3D). In contrast, we observed significant increase in E4orf6/7 protein levels of 187.2% and 145.7% after expression of PML-I or PML-II, respectively (Fig. 3E). Intriguingly, regulation of E4orf6 and E4orf6/7 protein expression by PML isoforms in H1299 cells differs from HepG2 cells. Here E4orf6/7 expression was not varying among the PML isoforms and we solely observed a significant decrease of 76.8% for E4orf6 (Fig. 3D) in PML-I expressing H1299 cells.

E4orf3 is the viral factor necessary for the reorganization of PML-NBs into tracks, mediated through its interaction with PML isoform II (29). While E4orf3 levels were not significantly changed by PML isoforms in H1299 cells, the expression of E4orf3 was significantly downregulated by 58.8% in HepG2 PML-IV and by 60.4% in HepG2 cells expressing PML-V (Fig. 3F).

The capsid-associated protein pVI stabilizes the viral capsid (72), promotes efficient viral replication by counteracting initial antiviral host responses through Daxx regulation (38) and disrupts the endosome after clathrin-mediated endocytosis (73, 74). Western blot analysis revealed distinct regulation of viral pVI in HepG2 and H1299 cells. While pVI was significantly reduced by PML-III (49.6%) and PML IV (55.5%) in HepG2 cells, we detected 215.5% and 202.8% upregulation of pVI levels by PML-II and PML-III in H1299 cells, respectively (Fig. 3G).

The viral packaging protein 1 (IVa2) is a component of the packaging machinery, which encapsidates the viral DNA and acts as a transcriptional activator of the viral major late promoter MLP (75–77). IVa2 protein levels in HepG2 cells were significantly decreased 40.2% by PML-II, 59.7% by PML-III, 66.2% by PML-IV, 45.5% by PML-V, and 35.7% by PML-VI compared with shPML cells. In H1299 cells IVa2 levels were only significantly reduced by 26.1% after PML-VI expression (Fig. 3H).

**FIG 4:**
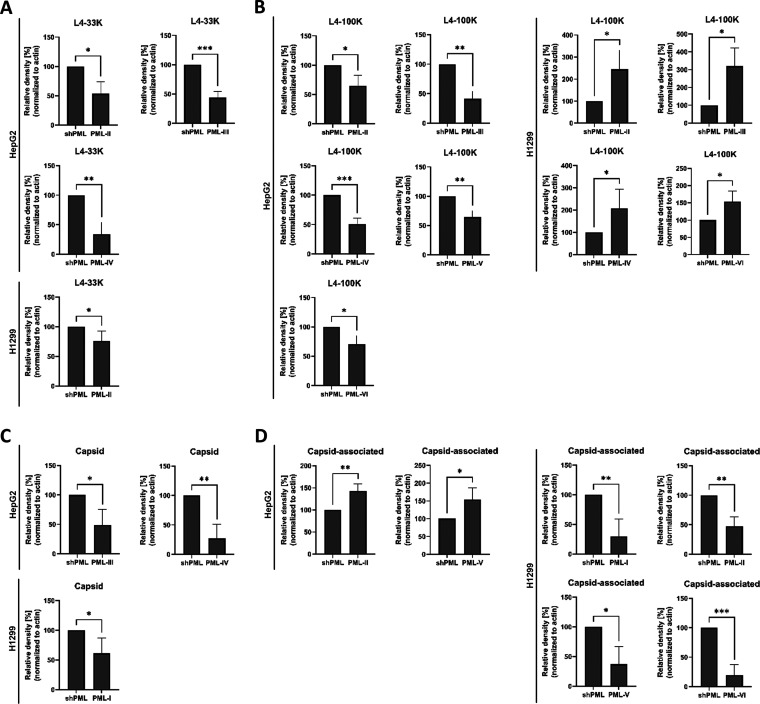
PML isoforms modulate late viral protein production. HepG2 shPML/EYFP-PML.n and H1299 shPML/EYFP-PML.n cells were infected with HAdV-wt at a multiplicity of 50 FFU/cell or 20 FFU/cell, respectively, and harvested 24h p.i. Total-cell lysates were prepared and proteins were separated using SDS-PAGE and detected via immunoblotting using anti-L4-33K, anti-L4-100K, L133 (anti-Capsid) and AC-15 (anti-β-actin). Densitometric analyses of detected protein levels quantified with *ImageJ* (version 1.51f) software and normalized to respective α-β-actin steady state levels. Bar charts represent average values and standard deviations based on three independent experiments in HepG2 cells and on four independent experiments for H1299 cells for (A) L4-33K, (B) L4-100K, (C) Capsid and (D) Capsid-associated. Statistically significant differences were assessed using an unpaired Student’s *t* test with the GraphPad Prism9 software. *, *P* ≤ 0.05; **, *P* ≤ 0.01; ***, *P* ≤ 0.001; ****, *P* ≤ 0.0001.

The late protein L4-33K is an adenoviral alternative RNA splicing factor that activates splicing of HAdV late gene transcripts and promotes viral packaging (78–81).In both cell lines L4-33K levels were significantly reduced after expression of PML-II by 46.6% in HepG2 cells and by 23.7% in H1299 cells. Additionally, we observed a significant reduction of 56.1% and 66.1% by PML-III and PML-IV, respectively (Fig. 4A). L4-100K inhibits host translation while promoting late viral translation (82). Beside viral translation, L4-100K is also involved in virion assembly and acts as a chaperone protein for hexon trimerization (83). Intriguingly, L4-100K expression was differentially modulated in HepG2 and H1299 cells because proteins levels of L4-100K were reduced in HepG2 cells, while increased in H1299 cells compared with the respective shPML control cell lines. L4-100K levels were significantly diminished 35.2% by PML-II, 58.8% by PML-III, 35.5% by PML-V, and 29.2% by PML-VI in HepG2 cells, whereas they were increased 291.1% by PML-II, 412.7% by PML-III, 237.6% by PML IV, and 113.6% by PML-VI in H1299 cells (Fig. 4B).

The expression of the major capsid proteins in HepG2 cells was significantly reduced by PML-III and PML-IV by 51% and 73.1%, respectively, compared with shPML cells, while PML-I reduced Capsid protein levels by 41.3% in H1299 cells (Fig. 4C). However, Capsid-associated proteins at around 20 kDa were significantly increased upon PML-II and PML-VI expression in HepG2 cells by 43% and 54.4%, respectively. In contrast, we observed significant decrease in Capsid-associated protein levels due to PML-I, PML-II, PML-V, and PML-VI expression by 70.2%, 52.3%, 62.9%, and 80% in H1299 cells, respectively (Fig. 4D).

### Disruption of PML-NB integrity by HAdV is highly dependent on PML isoforms.

**FIG 5:**
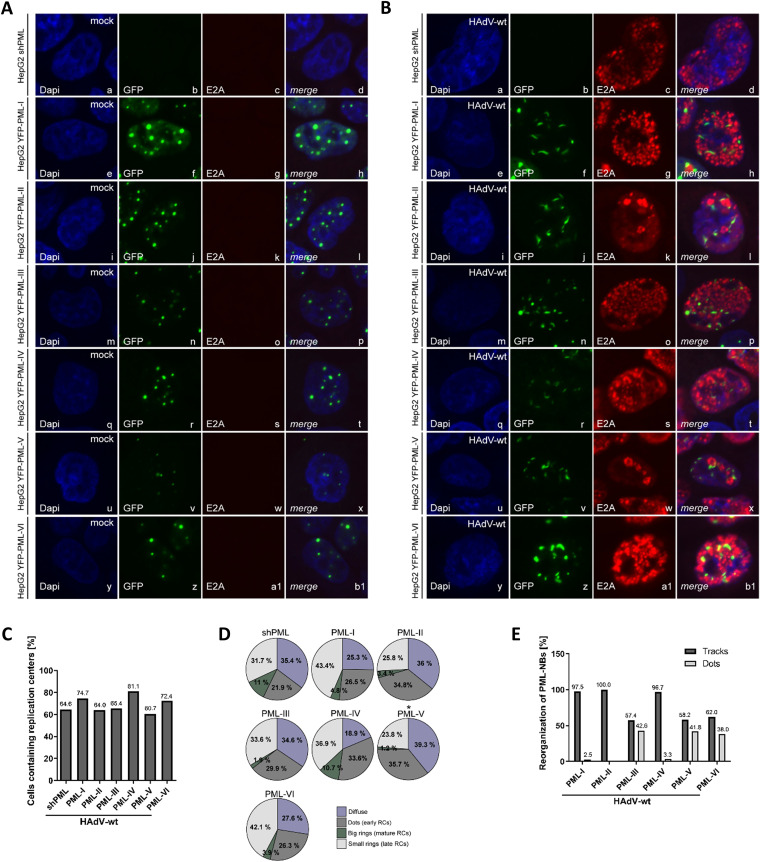
PML track and viral replication center formation differs among PML isoforms in HepG2 cells. (A, B) HepG2 shPML/EYFP-PML.n cells were either mock infected (A) or infected with HAdV-wt at a multiplicity of 50 FFU/cell (B). Cells were fixed with 4% paraformaldehyde (PFA) 24 h p.i. and double-labeled with MAb rabbit anti-GFP (PML isoforms) and MAb mouse B6-8 (anti-E2A). Primary Abs were detected with Alexa Fluor-488 anti-mouse (anti-GFP; EYFP-PML.n, green)- and Alexa Fluor-647 (E2A, red)-conjugated secondary Abs. Nuclear staining was performed using DAPI (4’,6-diamidino-2-phenylindole). Anti-GFP (green; panels b, f, j, n, r, v, z) and anti-E2A (red; panels c, g, k, o, s, w, a1) staining patterns representative of at least 50 analyzed cells are shown. Overlays of single images (merge) are shown in panels d, h, l, p, t, x, b1. (C, D) The formation of HAdV replication centers was monitored in at least 50 infected HepG2 shPML/EYFP-PML.n cells. (C) The bar chart represents the percentage of infected cells with replication centers as opposed to infected cells containing only diffuse E2A distribution. *P* values were calculated employing a Fisher exact test using the R language and environment for statistical computing. *, *P* ≤ 0.05; **, *P* ≤ 0.01; ***, *P* ≤ 0.001; ****, *P* ≤ 0.0001. (D) Pie charts represent percentage of infected HepG2 shPML/EYFP-PML.n cells containing specific type of replication centers. (E) PML-NB distribution into tracks was monitored in at least 50 infected HepG2 shPML/EYFP-PML.n cells. The bar chart shows the percentage of infected HepG2 shPML/EYFP-PML.n cells that were reorganized (tracks) and the percentage of infected HepG2 shPML/EYFP-PML.n cells without reorganization of PML-NBs (dots).

**FIG 6:**
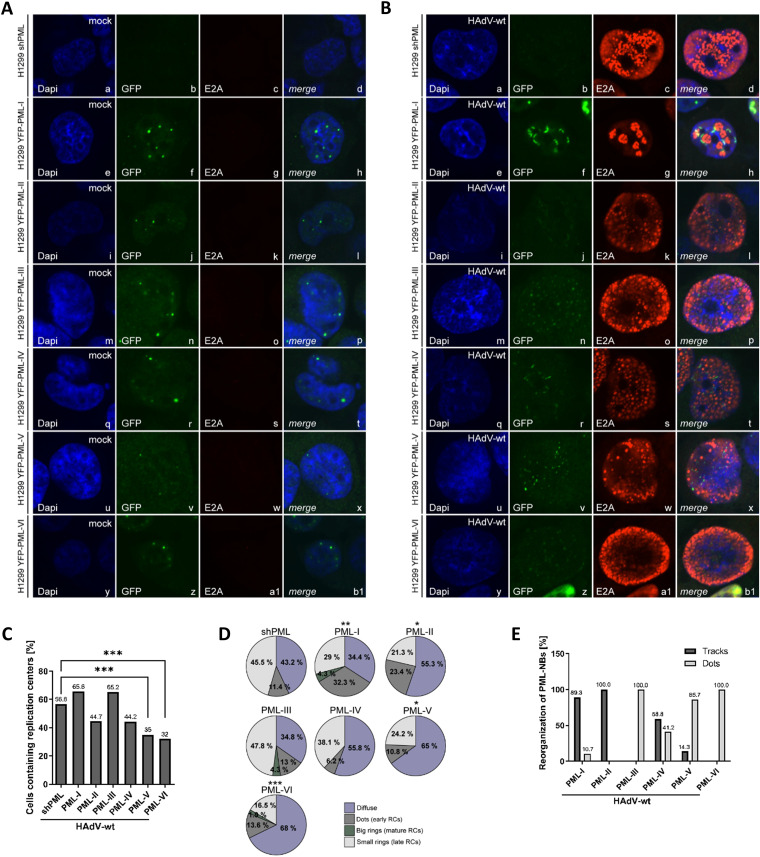
Disruption of PML-NBs and establishment of viral replication centers is regulated by PML isoform expression in H1299 cells. (A, B) H1299 shPML/EYFP-PML.n cells were either mock infected (A) or infected with HAdV-wt at a multiplicity of 20 FFU/cell (B). Cells were fixed with 4% paraformaldehyde (PFA) 24 h p.i. and double-labeled with MAb rabbit α-GFP (EYFP-PML.n) and MAb mouse B6-8 (anti-E2A). Primary Abs were detected with Alexa Fluor-488 α-mouse (α-GFP; EYFP-PML.n, green)- and Alexa Fluor-647 (E2A, red)-conjugated secondary Abs. Nuclear staining was performed using DAPI (4’,6-diamidino-2-phenylindole). Anti-GFP (green; panels b, f, j, n, r, v, z) and anti-E2A (red; panels c, g, k, o, s, w, a1) staining patterns representative of at least 50 analyzed cells are shown. Overlays of single images (merge) are shown in panels d, h, l, p, t, x, b1. (C, D) The formation of HAdV replication centers was monitored in at least 50 infected H1299 shPML/EYFP-PML.n cells. (C) The bar chart represents the percentage of infected cells with replication centers as opposed to infected cells containing only diffuse E2A distribution. *P* values were calculated employing a Fisher exact test using the R language and environment for statistical computing. *, *P* ≤ 0.05; **, *P* ≤ 0.01; ***, *P* ≤ 0.001; ****, *P* ≤ 0.0001. (D) Pie charts represent percentage of infected H1299 shPML/EYFP-PML.n cells containing specific type of replication centers. (E) PML-NB distribution into tracks was monitored in at least 50 infected H1299 shPML/EYFP-PML.n cells. The bar chart shows the percentage of infected H1299 shPML/EYFP-PML.n cells that were reorganized (tracks) and the percentage of infected H1299 shPML/EYFP-PML.n cells without reorganization of PML-NBs (dots).

To investigate the effects of PML isoforms on HAdV induced PML track formation, immunofluorescence studies were performed in infected HepG2 shPML/EYFP-PML.n (Table 1) (Fig. 5) and H1299 shPML/EYFP-PML.n (Table 2) (Fig. 6) cells. In non-infected cells, all PML isoforms showed the expected nuclear localization and are capable of forming nuclear bodies without the background of the additional PML isoforms (Fig. 5A, 6A) as it was previously published (29). Upon HAdV-wt infection the early viral protein E4orf3 interacts with PML-II mediating the disruption of the dot-like structure into PML tracks. However, those experiments were performed in PML^−/−^ mouse fibroblasts with transiently expressed individual FLAG-tagged PML isoforms under the control of a CMV promoter (29). In contrast, in this study comprehensive analysis was performed in stably transduced human hepatocellular or lung carcinoma cell lines expressing PML proteins. The limitation of our approach is that used shRNA knockdown method does not guarantee to eliminate all endogenous PML expression. Therefore, it is possible that PML-NB reorganization can in part be attributed to association of the introduced single YFP-tagged isoform with low levels of residual endogenous PML. However based our data (Fig. 1C, F) we are confident that the shRNA PML knockdown was highly efficient. Additionally, the study by Hoppe et al. (29), reported data originating from findings in cells transfected with HAdV5 E4orf3 plasmid. We performed our experiments in infected cells, as the data showing the influence of specific PML isoforms on track formation during HAdV infection have so far not been available.

In accordance to previously published findings (29), our immunofluorescence studies revealed a disruption of PML-NBs and formation of tracks in cells expressing PML-II. PML-NBs have been reorganized into tracks in 100% of PML-II expressing cells (Fig. 5B, panels j and l; Fig. 5E; Fig. 6B, panels j and l; Fig. 6E). Interestingly, our data also showed the reorganization of PML-NBs into tracks in cells expressing PML-isoforms other than PML-II (Fig. 5B, E; Fig. 6B, E). In particular, PML-NBs exclusively composed out of PML-I showed predominantly track-like distribution in 97.5% of HepG2 cells (Fig. 5B, panels f and h; Fig. 5E) and 89.3% of H1299 cells (Fig. 6B, panels f and h; Fig. 6E). PML-IV containing NBs were distributed into tracks in 96.7% of infected HepG2 cells (Fig. 5B, panels r and t; Fig. 5E), and 58.8% of H1299 cells (Fig. 6B, panels r and t; Fig. 6E). In HepG2 cells exclusively expressing PML-III, -V, or -VI, PML-NBs were reorganized into PML tracks in 57.4% for PML-III, 58.2% for PML-V, and 62% for PML-VI (Fig. 5B, panels n and p, v and x, z, and b1; Fig. 5E). On the other hand, in H1299 cells containing only PML-V, disruption of PML-NBs was downregulated to 14.3% (Fig. 6B, panels v and x; Fig. 6E). Remarkably, reorganization of PML-NBs into tracks by HAdVs was completely absent in H1299 cells expressing PML-III and PML-VI and all PML-NBs were still organized in dot-like structures during infection (Fig. 6B, panels n, p, z, and b1; Fig. 6E).

### Replication center formation varies among PML isoform expressing cells.

HAdV replication centers (RCs) localize next to PML-NBs on an E2A SUMOylation dependent manner (27). Here, we observed an isoform-specific regulation of RC formation. Dynamics of RCs during HAdV infection is tightly connected to the efficiency of viral replication. Therefore, we investigated in detail the influence of single PML isoforms on the abundance and morphology of RC. After staining for the early viral marker E2A/DBP, we observed that the appearance of E2A/DBP differs between the cell lines, each expressing a different PML isoform. Based on our findings, we classified the RCs into four phenotypes: diffuse, dots, big rings, and small rings. According to previous studies, the morphology of RCs classified as dots represents early HAdV RCs, big rings represent mature RCs, while late RCs appear as small rings (84). Our data did not show statistically significant differences in number of cells containing RCs in infected HepG2 cells expressing different PML isoforms (Fig. 5B, C). Additionally, morphology of viral RCs was moderately influenced by PML isoforms (Fig. 5D). Only in PML-V expressing HepG2 cells we observed statistically significant differences in RC morphology compared to shPML cells. Namely, in shPML cells we observed 35.4% cells with diffuse E2A distribution, 21.9% of cells contained early RCs, 11% of cells had mature RCs, and 31.7% cells late RCs. On the other hand, we found diffuse E2A distribution in 23.8% of PML-V expressing cells, 39.3% of cells contained early RCs, 35.7% of cells had mature RCs, and only 1.2% contained late RCs (Fig. 5D).

Influence of PML isoforms on RC formation was more pronounced in H1299 cells. The expression of PML-V and -VI significantly reduced the amount of cells containing RCs for 21.8% and 24.8%, respectively. However, the H1299 cells expressing PML-I, II, -III, and -IV did not show significant difference in RC formation compared to shPML cells (Fig. 6C). Besides the RC formation, PML isoforms have additionally more impact on RC morphology in H1299 cells. We observed statistically significant differences in RC morphology compared with shPML cells, in H1299 cells expressing PML-I, PML-II, PML-V, and PML-VI. In particular, we found diffuse E2A distribution in 43.2% of shPML cells, 34.4% of PML-I expressing cells, 55.3% of PML-II cells, 65% of PML-V cells, and 68% of PML-VI expressing H1299 cells. Early stages of RCs were found in 11.4% of H1299 shPML cells, in 32.3% of cells expressing PML-I, in 23.4% expressing PML-II, in 10.8% expressing PML-V and in 13.6% expressing PML-VI (Fig. 6D). There were no mature RCs present in shPML, PML-II, and PML-V expressing H1299 cells, while mature RCs were found in 4.3% of PML-I and 1.9% of PML-VI expressing H1299 cells (Fig. 6D). Late RCs were observed in 45.5% of shPML cells, 29% of PML-I expressing cells, 21.3% of PML-II cells, 24.2% of PML-V cells, and in 16.5% of PML-VI expressing H21299 cells (Fig. 6D).

## DISCUSSION

PML-NBs and their components play a crucial role in antiviral defense and are therefore, targeted by many viruses (29, 37, 39, 85). Simultaneously, several PML-NBs associated proteins are also identified to promote viral replication (21, 22, 27, 33, 40). HAdV reorganizes PML-NBs into track-like structures antagonizing their antiviral activity, while beneficial components of the PML-NBs become accessible for viral determinants (29, 37, 39, 85). Here we performed in depth functional analysis of nuclear PML isoforms I-VI during HAdV infection.

Our data showed that PML-I promotes viral replication in HepG2 cells (Fig. 2A). PML-I expression was sufficient for efficient RC formation (Fig. 5C, D), even though it led to the reduction of E2A and E4orf6 protein levels (Fig. 3C, D). On the other hand, PML-I led to the increase in E4orf6/7 protein levels (Fig. 3E). Even though these changes indicate a role of PML-I in the regulation of specific viral protein synthesis, they are not sufficient to explain the promoting effect of PML-I expression on virus infection. However, a conclusion that arose from our findings was that PML-NBs composed out of PML-I can also be reorganized into the PML-tracks during HAdV infection and in the extent comparable with that in PML-II expressing cells (Fig. 5B, E). Up to now the dogma is that HAdV reorganize PML-NBs through PML-II, specifically via the interaction of viral E4orf3 with PML-II. Experiments by Hoppe et al. (29) showing this, were performed in PML negative primary mouse embryo fibroblasts (MEFs) with a transient transfection of the single PML isoforms. These constructs contain a CMV promoter, which might be additionally induced by early adenoviral proteins (86). In contrast, experiments in this study were performed during infection of human mammalian cell lines with a stable expression of the different human PML isoforms under the control of the HSV-1 gD promoter. Therefore, we were able to acquire evidence that PML-NBs in cells without PML-II expression can be targeted by HAdV for disruption of PML-NB integrity, possibly through the mediation of another viral protein than E4orf3 (Fig. 5E and Fig. 6E). HAdV reorganize PML-NBs into tracks to inhibit their antiviral capacity and to simultaneously exploit their infection promoting components, a process being essential for efficient viral replication (22, 33, 87–91). Therefore, the ability of the virus to efficiently generate PML-tracks composed out of PML-I provides the explanation for PML-I infection promoting effect in HepG2 cells. In concordance with these findings, efficient viral RC formation and disruption of PML-NBs in PML-I expressing cells was also observed in H1299 cells (Fig. 6B, C, E). However, we could not detect a promoting role of PML-I in viral progeny production in H1299 cells (Fig. 2B). PML-I expression in H1299 cells increased E1A protein levels (Fig. 3A). Even though E1A has the ability to induce the expression of other viral proteins, this was not the case in PML-I expressing cells. Our data even showed that PML-I reduced the expression of viral proteins E4orf6, Capsid and Capsid-associated proteins (Fig. 4C, D). It is possible that in H1299 cells, positive effects of formed PML-tracks are neutralized by a PML-I inhibitory effect on late protein synthesis resulting in the lack of effect of PML-I on viral progeny production observed in our study. PML acts as an essential regulator of p53 activity through sequestering MDM2 to the nucleolus and thus mediating destabilization of p53 (44). Reports suggest that PML-I is the splice variant, which is efficiently recruited to the nucleolus and crucial for nucleolar targeting of the other endogenous PML isoforms (92). Because H1299 cells are p53-negative, HAdV are not reliant on efficient targeting of p53, whereby PML-I supportive function might be more prominent in p53-positive HepG2 cells.

We have recently reported that PML-II enhances viral progeny production compared to that in the parental cell line (32). In line with these facts, our data showed that in H1299 cells PML-II positively regulates HAdV replication (Fig. 2B). Intriguingly, PML-II expression does not affect viral progeny production in HepG2 cells (Fig. 2A), indicating once again the cell line specific behavior of PML isoforms. The differences we observed are not unexpected as we performed our experiments in cells of either lung or liver origin and it is known that PML composition and expression highly depends on cell type, cell cycle and transformation (3, 93). In both cell lines all PML-NBs are disrupted and reorganized in PML-II expressing cells, whereby antiviral mechanisms are counteracted by HAdV and positive host factors are accessible to ensure viral replication (Fig. 5 and 6E) (22, 29, 32, 33, 37, 39, 40, 85). The analysis of protein expression during HAdV infection provides the reason underlying the inability of PML-II to promote viral replication in HepG2 cells. Namely, the expression of PML-II caused the reduction of L4-100K protein levels (Fig. 5A, B). As L4-100K performs the crucial role in translation of HAdV proteins (94, 95), it is not surprising that PML-II reduced the levels of several additional HAdV proteins (Fig. 3 and 4). It is possible that this defect neutralizes positive effect of PML-NB reorganization in tracks, rendering PML-II expression insignificant for viral replication.

It has been reported that PML-III restricts replication of many RNA and DNA viruses, such as Influenza A, HFV (human foamy virus), VSV (vesicular stomatitis virus) (26, 96). In accordance, here we show that PML-III prevents HAdV progeny production in H1299 cells (Fig. 2B), with a severe negative effect on PML track formation (Fig. 6E). Even though PML-III exhibits overall inhibitory effect on viral replication, its expression increased viral mRNA and protein expression in H1299 cells. On the other hand, in HepG2 cells, PML-III showed inhibitory effect on viral protein synthesis, but did not affect viral replication. This can be explained by the finding that the virus was able to reorganize PML-NBs in 57.4% of HepG2 cells (Fig. 5E) and therefore counteract host antiviral mechanisms to ensure efficient viral progeny production (Fig. 2A). These findings show cell line specific influence of PML-III on HAdV life cycle. Additionally, the molecular weight of PML-III is approximately 20 kDa higher in HepG2 cells compared to H1299 cells (Fig. 1A, C). Since, PML-III has a weight of around 70 kDa (12), PML-III detected in HepG2 cells appears to be a posttranslational modified moiety (43). Although the molecular mechanism has so far remained elusive, it seems that posttranslational modification of PML-III differs in the distinct tissues modulating its function in regulation of HAdV infection and replication. Common conclusion from these studies is that PML-NBs reorganization has a determining role during infection. Namely, the increased levels of viral proteins in PML-III expressing H1299 cells were completely unable to counteract the overpowering negative influence of the virus inability to reorganize PML-III containing PML-NBs. On the other hand, the defect in viral protein synthesis in HepG2 cells was fully compensated by even partial PML-NB reorganization into track-like structures.

Besides PML-III, PML-IV is also known to restrict viral infections, such as VZV, EMCV, and rabies virus, either through a so far unknown mechanism (97) or by simply sequestering viral proteins to the PML-NBs (98, 99). Our studies suggest a negative regulation of HAdV infection by PML-IV in H1299 cells (Fig. 2B). Intriguingly, neither adenoviral DNA synthesis, mRNA expression or HAdV RCs formation are affected by PML-IV. Additionally, PML-IV even increases L4-100K protein synthesis in H1299 cells (Fig. 4B). Even though the expression of L4-100K is beneficial for the infection, the virus is unable to fully relocalize PML-NBs into tracks (Fig. 6E). This could offer the explanation for the inhibitory effect of PML-IV. On the other hand, in HepG2 cells, the virus was able to efficiently reorganize PML-IV containing PML-NBs into tracks (Fig. 5E). This led to the inability of the cell to restrict HAdV infection, even though PML-IV exhibited a strong negative effect on HAdV protein synthesis. Together, these findings emphasize once more the importance of the loss of PML-NB integrity for productive HAdV infection.

Furthermore, our studies provide evidence for an inhibitory effect of PML-V on HAdV infection in HepG2 cells (Fig. 2A). PML-V expression caused the reduction of E4orf6, E4orf3, IVa2, and L4-100K protein levels (Fig. 3D, F, H; Fig. 4B). Even though the total number of HAdV RCs was not affected by PML-V, maturation of RCs was slowed down (Fig. 5D). Additionally, HAdV was unable to fully reorganize PML-tracks (Fig. 5E). Together, these findings provide a clear explanation for PML-V mediated inhibition of HAdV replication in HepG2 cells. Although HAdVs did not form proper viral RC (Fig. 6C, D), nor did they fully disrupt PML-NBs (Fig. 6E) in H1299 cells expressing only PML-V, the viral progeny production remained undisrupted (Fig. 2B). Next to the fact that the expression of the majority of examined viral proteins was not affected by PML-V (Fig. 3 and 4), it is possible that PML-V has so far undiscovered strong positive impact on viral replication that compensates defect in PML-track formation. However, the precise answer to this question requires further studies.

PML-VI isoform is shows restrictive activity toward HIV-1 (100) and certain influenza A strains (101). In concordance with these studies, we found that PML-VI was the only PML isoform showing the clear negative effect on HAdV replication in both tested cell lines. The expression of PML-VI significantly reduced viral progeny production (Fig. 2A, B). Intriguingly, PML-VI caused the reduction of IVa2 protein levels in both cell lines (Fig. 3H), indicating the possible impact on HAdV genome packaging controlled by IVa2 (63, 102). Similar to PML-V, RC establishment in H1299 cells was impaired (Fig. 6C, D) and PML-NBs composed of PML-VI were not disrupted (Fig. 6E), and thus antiviral defense response of the host cell was not targeted to establish efficient viral replication. Next to the reduction of protein levels of certain HAdV proteins, even the partial defect in establishment of PML-tracks in HepG2 cells was enough to allow negative PML-VI influence on viral production.

In summary, PML isoform behavior is cell line specific, and thus differentially regulates productive HAdV replication and progeny production. Here we show for the first time, that HAdV can reorganize PML-NBs composed out of PML isoforms other then PML-II. Our findings clearly illustrate the importance of PML-track formation for efficient viral production. Namely, PML-NBs composed out of the PML isoforms with negative influence on the viral replication were unable to be fully disrupted into tracks during HAdV infection. On the other hand, PML-NBs composed out of PML isoforms with a beneficial influence on the virus formed tracks in all examined cells. In sum, we provide novel insight into virus induced PML track formation, PML-isoform-mediated RC formation and PML-isoform-specific regulation of viral replication and progeny production.

## MATERIALS AND METHODS

### Cell culture.

HEK293 (ECACC European Collection of Authenticated Cell Cultures; Sigma-Aldrich, No. 85120602-1VL), HepG2 shPML, HepG2 EYFP-PML.n (PML-I to PML-VI), H1299 shPML and H1299 EYFP-PML.n (PML-I to PML-VI) cells were cultivated as a monolayer with Dulbecco's Modified Eagle Medium (DMEM) containing 0.11 g/l sodium pyruvate, 5% fetal calf serum (FCS) for 293 and H1299 cells, 10% FCS for HepG2, and 1% Penicillin/Streptomycin (100 U/mL penicillin and 10 mg/mL streptomycin in 0.9% NaCl) in a 5% CO_2_ atmosphere at 37°C. Lentivirus transduced cells were maintained with continuous antibiotic selection, as appropriate.

### Cell viability assay.

Cell viability in response to HAdV infection and expression of single PML isoforms was measured 24 h to 48 h postinfection using the Promega (Madison, WI) CellTiter-Blue Cell Viability Assay system according to the manufacturer’s protocol. Fluorescence values were measured using a Tecan (Männedorf, Switzerland) Infinite 200M plate reader.

### Lentivirus transduction.

Lentivirus supernatants were prepared from HEK293 cells after co-transfection of a lentivirus vector plasmid with pVSV-G (expressing the VSV envelope protein) and pCMV.DR8.91 (expressing lentivirus helper functions). Supernatant was sterile-filtered (0.22 μm) and stored at −80°C after adding 10% glycerine. HepG2 (103) shPML and H1299 (ATCC, No. CRL-5803) shPML cells were generated by using lentiviral vector based on pLKO.1 puro encoding a short hairpin RNA (shRNA) against codons 394 to 400 in PML exon 4 which is conserved in all major PML isoforms as described in Everett et al. (21). The sense-strand DNA sequence of the anti-PML shRNA was 5′-AGATGCAGCTGTATCCAAG-3′. Stable cell lines were then selected with puromycin (initially 2 μg/mL, then reduced to 1 μg/mL during subsequent passage).

Generated cell lines were subsequently transduced with lentiviruses derived from pLKO.1puro vectors (pLKOneo-gD-EYFP-PML.n) encoding for N-terminal enhanced yellow fluorescent protein-tagged (EYFP) human PML isoforms named according to the nomenclature of Jensen et al. as PML-I (AAG50180), PML-II (AF230410), PML-III (S50913), PML-IV (AAG50185), PML-V (AAG50181), and PML-VI (AAG50184), resistant to the shRNA by introduction of five silent point mutations in the relevant sequence kindly provided by Prof. Roger Everett (Glasgow) (43). Generated cell lines expressing EYFP-PML.n were selected with G418 (initially 2 mg/mL, then reduced to 1 mg/mL during subsequent passage).

### Human adenoviruses.

H5*pg*4100 served as the wild-type (wt) HAdV5 parent virus in these studies. Viruses were propagated and titrated in HEK293 monolayer cells by fluorescent focus assay as described previously (50). To measure viral progeny production cells were harvested 24 h (p.i.) and lysed by three cycles of freeze and thaw. The cell lysates were serially diluted in DMEM for infection of HEK293 cells and virus yield was determined by quantitative immunofluorescence staining of E2A-DBP protein 24 h p.i. as previously published (104). Viral DNA replication was monitored using quantitative PCR. Therefore, cells were lysed by RIPA buffer as previously described (105) and viral DNA was isolated by proteinase K digest. Samples were diluted 1:200 in nucleic acid-free water (Promega) and 5 μL were subjected to qPCR analysis using the appropriate primer: actin fwd, 5′-CTTCGCGGGCGACGAT-3′; rev, 5′-CCACATAGGAATCCTTCTGACC-3′; Hexon fwd primer, 5′-CGCTGGACATGACTTTTGAG-3′; Hexon rev primer, 5′-GAACGGTGTGCGCAGGTA-3′.

### Antibodies and protein analysis.

For protein analysis, cells were resuspended in RIPA lysis buffer (50 mM Tris-HCl [pH 8,0], 150 mM NaCl, 5 mM EDTA, 1% [vol/vol] Nonident P-40, 0.1% [wt/vol] SDS, 0.5% [wt/vol] sodium deoxycholate) supplemented with a fresh protease inhibitor cocktail containing 0.2 mM PMSF, 1 mg/mL pepstatin A, 5 mg/mL aprotinin, and 20 mg/mL leupeptin. After 30 min incubation on ice, the lysates were sonicated (40 pulses; output 0.80; 0.8 impulse/s) and cell debris was pelleted at 14,800 *g* at 4°C for 3 min. Cell lysates were boiled for 3 min at 95°C in 5x laemmli buffer and analyzed by immunoblotting.

Primary antibodies specific for HAdV proteins used in this study included mouse monoclonal antibody (MAb) mouse M73 (anti- E1A) (106), MAb mouse 2A6 (anti- E1B-55K) (107), MAb mouse B6-8 (anti-E2A) (108), pAb rabbit anti-Pol (109), MAb mouse RSA3 (anti-E4orf6) (110), MAb rat 6A11 (anti-E4orf3) (70), polyclonal antibody (pAb) rabbit pVI (74), MAb rabbit pVII (74), pAb rabbit L4-33K (kindly provided by Prof. Pat Hearing), MAb rat L4-100K (111), pAb rabbit anti-IVa2 (kindly provided by Prof. Pat Hearing) (112), and Ad polyclonal rabbit serum L133 (50). Primary antibodies specific for cellular and ectopically expressed proteins included pAb rabbit PML (ab72137; Abcam), MAb mouse AC-15 (anti-β-actin; Sigma-Aldrich, Inc.) and a pAb rabbit anti-GFP epitope MAb (ab290, Abcam).

Secondary antibodies conjugated to horseradish peroxidase (HRP) for detection of proteins by immunoblotting were anti-rabbit IgG, anti-mouse IgG, and anti-rat IgG (Jackson/Dianova) used in a dilution of 1:10,000.

### Indirect immunofluorescence.

For indirect immunofluorescence analysis cells were grown on glass coverslips. At the indicated times cells were fixed for 10 min using 4% paraformaldehyde and permeabilized in 0.5% Triton X-100 for 5 min on room temperature. Coverslips were blocked for 1 h in TBS-BG buffer (20 mM Tris-HCl [pH 7.6], 137 mM NaCl, 3 mM KCl, 1.5 mM MgCl_2_, 0.05% [vol/vol] Tween 20, 0.05% [wt/vol] Na-acid, 5% [wt/vol] BSA, 5% [wt/vol] Glycine) followed by incubation in primary antibodies diluted in PBS for 1 h to detect the desired antigens. After washing three times in TBS-BG buffer, cells were treated with DAPI and the corresponding Alexa647/Alexa488-conjugated secondary antibodies (Dianova/Invitrogen). Coverslips were washed three times in TBS-BG, mounted in mowiol and digital images were acquired using a *Nikon TiE* microscope equipped with *Perkin Elmer UltraView Vox System*. Images were analyzed using Volocity software and cropped using Adobe Photoshop CS4 and assembled with Adobe Illustrator CS4.

### HAdV RNA and DNA synthesis.

Subconfluent H1299 and HepG2 shPML and EYFP-PML.n cells were infected with wild-type virus at a multiplicity of 20 FFU/cell or 50 FFU/cell, respectively. Cells were harvested 24 h p.i. followed by total RNA isolation with TRIzol reagent (Thermo Scientific) according to the manufacturer’s protocol. Amount of RNA was measured and 1 μg of RNA was reverse transcribed according to the manufacturer’s protocol (Promega Reverse Transcription System). For qPCR the following primer were used to amplify specific viral RNA and DNA: SimpleChIP Human RPL30 Exon 3 Primers #701418S (Cell Signaling); 18S rRNA fwd primer, 5′-CGGCTACCACATCCAAGGAA-3′; 18S rRNA rev primer, 5′-GCTGGAATTACCGCGGCT-3′; CD45 (HTH6) fwd primer, 5′-GATTGACTACAGCAAAGATGC-3′; CD45 (HTH6) rev primer, 5′-TTGTGGTCTCTGAGAAGTCA-3′; IQGAP3 fwd primer, 5′-TTGTAGTTCCCGGCCGCGGAG-3′; IQGAP3 rev primer, 5′-ACAAGACGAGGAACTGCTGTAAGGG-3′; E1A fwd primer, 5′-GTGCCCCATTAACCAGTTG-3′; E1A rev primer, 5′-GGCGTTTACAGCTCAAGTCC-3′; Hexon fwd primer, 5′-CGCTGGACATGACTTTTGAG-3′; Hexon rev primer, 5′-GAACGGTGTGCGCAGGTA-3′.

Quantitative RT-PCR was performed in a LightCycler 480 Instrument II (Roche) or qTOWER^3^ (Analytik Jena) with a first-strand method. Five μL of a 1/10 cDNA template dilution (for detection of RNA) or 5 μL of a 1/200 proteinase K digest (for detection of DNA) was mixed with 10 pmol/μL of each synthetic oligonucleotide primer and 5 μL of LightCycler 480 Sybr green I Master (Roche) or Luna Universal qPCR Master Mix (NEB) per sample in a 96-well plate (FrameStar) 480/96 (4titude) or 96-well 0.2-mL 8-transformer plate (Biozym Scientific). The PCR conditions were set as follows: 10 min at 95°C followed by 40 cycles of 30 s at 95°C, 30 s at 62°C, and 30 s at 72°C. The average threshold cycle (Ct) value was calculated from triplicate reactions, and levels of adenoviral mRNA relative to RPL30, or adenoviral DNA relative to actin. The identities of the products obtained were confirmed by melting curve analysis.

### Statistical analyses.

Testing for statistically significant differences in mean values was performed using a one-way ANOVA, two-way ANOVA, unpaired Student’s *t* test, and Fisher’s exact test with the GraphPad Prism 5, GraphPad Prism9 and R software.
