# Supplementary material for: PML Alternative Splice Products Differentially Regulate HAdV Productive Infection
Source: Microbiol Spectr. 2022 Jun 14;10(4):e00785-22. doi: 10.1128/spectrum.00785-22 (PMC9431499; doi:10.1128/spectrum.00785-22)
Supplement: Supplemental file 1 — Fig. S1. Download spectrum.00785-22-s0001.pdf, PDF file, 2.8 MB [file spectrum.00785-22-s0001.pdf]

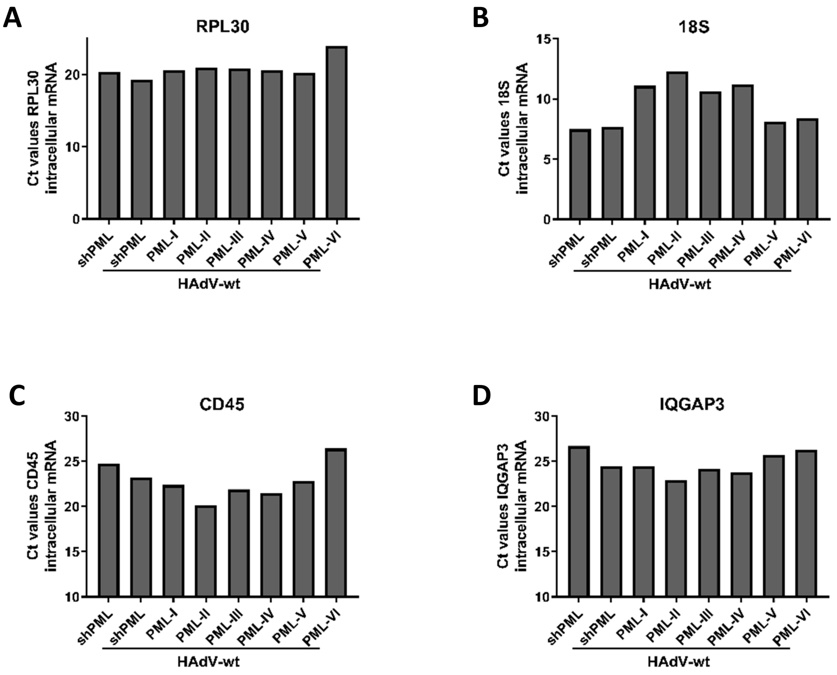
A
RPL30
Ct values RPL30 intracellular mRNA
0
10
20
shPML
shPML
PML-I
PML-II
PML-III
PML-IV
PML-V
PML-VI
HAdV-wt
B
18S
Ct values 18S intracellular mRNA
0
5
10
15
shPML
shPML
PML-I
PML-II
PML-III
PML-IV
PML-V
PML-VI
HAdV-wt
C
CD45
Ct values CD45 intracellular mRNA
10
15
20
25
30
shPML
shPML
PML-I
PML-II
PML-III
PML-IV
PML-V
PML-VI
HAdV-wt
D
IQGAP3
Ct values IQGAP3 intracellular mRNA
10
15
20
25
30
shPML
shPML
PML-I
PML-II
PML-III
PML-IV
PML-V
PML-VI
HAdV-wt

Supplementary Figure.

Steady state levels of RPL30 mRNA were shown to be stable among cells expressing single PML isoforms. H1299 shPML / EYFP-PML.n cells were infected with HAdV-wt at a multiplicity of 20 FFU/cell and harvested 24 h p.i.. Total mRNA was isolated using TRIzol, reverse transcribed and analyzed by RT-qPCR using primers specific for (A) RPL30, (B) 18S, (C) CD45, and (D) IQGAP3. Bar charts represent average values of technical duplicates.
